# Cellular mechanisms of inflammation

**DOI:** 10.1080/09629359791866

**Published:** 1997-04

**Authors:** G. Camus

**Affiliations:** 1 Centre Interdisciplinaire de Biochimie de l'Oxygène Institut de Chimie B6, Sart Tilman Liège 4000 Belgium


Mediators of Inflammation, 6, 154–158 (1997)

### Cellular mechanisms of inflammation

G. Camus

Centre Interdisciplinaire de Biochimie de l'Oxygène, Institut de Chimie, B6, Sart Tilman, 4000 Liège, Belgium
Tel: (+32) 4 3663360
Fax: (+32) 4 3662866

### Introduction

The second meeting of the contact group placed under the patronage of the Fonds National de la Recherche Scientifique (FNRS, Belgium) and entitled 'Cellular Mechanisms of Inflammation' held in Yvoir, Belgium, on 11 October 1996. This meeting began with the master lecture by Dr R. Monteiro (INSERM, Paris) on the potential role of the IgA Fc receptors (FcαR) in the catabolism of IgA. This set the stage for communications by Godding *et al.* and Hoang *et al.* dealing with the role on interferon gamma on the production of secretory IgA by human bronchial epithelial cells *in vitro* and the regulation of cytokine production by human mononuclear cells of the lamina propria, respectively. Results from research on the production of cytokines by human chondrocytes (Henrotin *et al.*) and during human muscular exercise (Croisier *et al.*) were presented. Courtois *et al.* described the purification of a NADH-hypothiocyanite-oxidoreductase in *Streptococcus sanguis*. Mathy-Hartert *et al.* documented the protective effect of ceftazidime on human endothelial cells damaged by anoxia-reoxgygenation *in vitro*. Various aspects of neutrophil functions were studied *in vitro* by Mouithys-Mickalad *et al.* and in trauma patients by Deby-Dupont *et al.*

We are happy to present the results of these experimental studies in the current issue of *Mediators of Inflammation*.

This meeting has benefited from the assistance of the Fonds National de la Recherche Scientifique (FNRS, Belgium). We are deeply grateful to all the contributors.

### Secretory component (SC) production by human bronchial epithelial cells is upregulated by interferon gamma (IFN-γ)

V. Godding,[1,2] Y. Sibille,[1,2] M. Delos,[1] D. Giffroy,[2] A. Langendries[2] and J. P. Vaerman[2]
[1]UCL Mont-Godinne, 5530 Yvoir and
[2]Experimental Medicine, ICP, UCL, 1200 Brussels, Belgium

Secretory IgA (SIgA) is the major immunoglobulin present in human respiratory secretions. Secretory component (SC) production by the epithelial cell regulates the transcytosis of IgA and its release as SIgA at the apical pole of the epithelial cell. We studied SC production and transcytosis by cultured HBEC monolayers. HBEC were isolated from healthy segments of bronchial tubes obtained from thoracic surgery. In primary culture on collagen-coated plates, HBEC production of SC was upregulated by IFN-γ 100 U/ml at 48 and 72 h ($4.5 \pm 4.1$ ng/ml/$10^5$ cells vs $8.9 \pm 7.1$ ng/ml/$10^5$ cells at 48 h; 5.7 ng/ml/$10^5$ cells $\pm$ 3.7 ng/ml/$10^5$ cells vs $13.1 \pm 7.3$ ng/ml/$10^5$ cells at 72 h, $p < 0.01$) ($n = 8$). In polarized cultures, HBEC demonstrated apically polarized SC production upregulated by IFN-γ at 48 and 72 h ($4.1 \pm 1.1$ ng/ml/$10^5$ cells vs $12.7 \pm 5.4$ ng/ml/$10^5$ cells at 48 h; $3.1 \pm 0.3$ ng/ml/$10^5$ cells vs $11.1 \pm 2.3$ ng/ml/$10^5$ cells at 72 h, $p < 0.02$) ($n = 4$). Furthermore, polarized HBEC demonstrated transcytosis of $^{125}$I-dimeric IgA at 24, 48 and 72 h ($n = 5$). Again, this process was upregulated by IFN-γ 100 U/ml at 48 and 72 h ($5.1 \pm 2.2$ ng*IgA/ml vs $10.1 \pm 0.6$ ng*IgA/ml at 48 h and $6.1 \pm 1.45$ ng*IgA/ml vs $10.9 \pm 0.8$ ng*IgA/ml at 72 h,

© 1997 Rapid Science Publishers

$p < 0.03$) ($n = 3$). An ELISA assay identified the IgA present in the apical media as SIgA.

We conclude that HBEC are capable of SC production and dIgA transcytosis *in vitro*. These processes are upregulated by IFN-$\gamma$.

### Markers of early neutrophil activation in plasma of trauma patients

G. Deby-Dupont, M. E. Faymonville, A. Adam, P. Damas and M. Lamy

Service d'Anesthésie-Réanimation, Centre Hospitalier Universitaire, B35, Domaine Universitaire du Sart Tilman, 4000 Liège, Belgium;

Faculté de Pharmacie, Université de Montréal, Québec, Canada

In early post-traumatic phase, the complement fragments released by the activation of the classical and alternative pathways play a pivotal role early in trauma by increasing permeability and activating phagocytic cells. These cells undergo respiratory burst and release a variety of inflammatory mediators and enzymes, capable of producing local cellular and tissue damages, but also of spreading the inflammatory reaction to other organs leading to the development of a systemic inflammatory reaction and multiple organ failure. In 15 patients (12 men, 3 women; mean age: $42 \pm 16$ years) with severe polytrauma (at least two major injuries to chest or abdomen and limbs) and a mean Injury Severity Score of $45 \pm 14$, we studied the evolution of the plasma concentrations of three markers of neutrophil activation, myeloperoxidase (MPO) and elastase (two specific enzymes from azurophilic granules), and lactoferrin (released from specific granules) from the first hour until 84 h after trauma. Nine of these patients developed ARDS (five within 24 h) and 10 presented septic complications after 2 to 6 days. High mean values of the three enzymes were observed in the first hours after trauma: 1605 ng/ml for MPO after 1 h, 1046 ng/ml for elastase after 6 h and 77 ng/ml after 12 h for lactoferrin (control values: $320 \pm 80$, $97 \pm 25$ ng/ml and $16 \pm 5$ ng/ml for MPO, elastase and lactoferrin respectively). MPO returns to normal values 30 h after trauma, while elastase and lactoferrin decrease but remain above normal values until 84 h after trauma. Uptake of MPO by monocytes or macrophages could explain this rapid disappearance from plasma. These observations firmly establish that multiple trauma induces a rapid and intense activation of phagocytic cells, as a consequence of both complement activation and direct stimulation. This early stimulation can be responsible for an explosive chain reaction resulting in subsequent multiple organ failure.

### Human colonic intraepithelial lymphocytes (IEL) regulate the cytokine production by lamina propria mononuclear cells (LPMNC)

P. Hoang,[1,2] J. P. Dehennin,[1,2] Li Li,[1] A. Geubel[1] and J. P. Vaerman[2]
[1]University of Louvain, Gastroenterology Unit, and [2]Experimental Medicine Unit, Institute of Cell Pathology, Brussels, Belgium

Ninety to 95% of intraepithelial lymphocytes (IEL) are T cells ($CD3^+$) and of these, approximately 80% are of the CD8 phenotype. Although their precise role *in vivo* remains enigmatic, it has been shown *in vitro* that under particular conditions human IEL are able to play some regulatory functions. It is believed that they may communicate with the adjacent epithelial cells and lamina propria cells. As an example, we have previously shown that colonic human IEL can inhibit the proliferation of autologous lamina propria mononuclear cells (LPMNC). IEL were also able to suppress IgA synthesis. Inhibitory mechanisms were shown to occur through a soluble factor, possibly a cytokine.

The aim of the study was to test the ability of colonic human IEL to produce interleukin-2 (IL-2), interferon-gamma (IFN-gamma) and interleukin-10 (IL-10). Furthermore, the putative immunoregulatory function of IEL on cytokine secretion by autologous LPMNC was examined.

Mucosal lymphocytes were obtained from colonic resection specimens. To study the immunoregulatory activity of IEL, $10^6$IEL were cultured with $10^6$LPMNC for 72 h with or without phytohaemagglutinin A (PHA). The control cultures consisted of IEL or LPMNC alone with or without PHA. Concentrations of IL-2, IFN-gamma and IL-10 in culture supernatants were measured using an amplified sensitivity immune assay.

Following stimulation with PHA, the median IL-2 production by IEL and LPMNC cultured alone was respectively 2.5 U/ml (range 1–26) and 9 U/ml (range 2–450), the median IFN-gamma production was 27 U/ml (range 1–105) and 26.5 U/ml (range 2–242), the median IL-10 production was 3 U/ml (range 1–10) and 4 U/ml (range 1–100). Co-culture experiments (LPMNC and IEL) showed that IEL affected only minimally IL-10 production, the median IL-10 production being 7.5 U/mol (range 2–340). In contrast, they enhanced the IL-2 and IFN-gamma production by LPMNC. After 3 days of co-culture, IL-2 and IFN-gamma production was respectively 38.5 U/ml (range 8–450) and 104 U/ml (18–500).

These data show that colonic IEL are able to produce IFN-gamma, IL-2 and IL-10 following stimulation with PHA even if the absolute amounts of cytokines produced were small. Furthermore IEL can enhance the PHA-induced synthesis of IL-2 and interferon-gamma by LPMNC but not that of IL-10. This phenomenon may prove of importance in the modulation of subsequent immunopathological reactions including both activation of inflammatory cells and the expression of class II expression molecules on epithelial cells.

### Effects of training on myocellular enzyme leakage and delayed onset muscle soreness following maximal isokinetic eccentric exercise

J-L. Croisier, G. Camus, J. Duchateau, G. Deby-Dupont, A. Juchmès-Ferir, I. Venneman, J-M. Crielaard, F. Thiry, C. Deby and M. Lamy
Centre for the Biochemistry of Oxygen, Institute of Chemistry, Sart Tilman, 4000 Liège, Belgium

To address the question of whether the training-induced reduction of delayed onset muscle soreness (DOMS) and musle damage caused by maximal eccentric contractions could be explained by a decrease of the inflammatory response to damaging contractions, 10 moderately active male volunteers were randomly assigned to two age-matched groups: a control group (CG; $n = 5$) and a trained group (TG; $n = 5$). All subjects were submitted to two isokinetic exercise sessions in the eccentric mode consisting of three stages of 30 maximal contractions of the knee extensor and flexor muscle groups of both legs separated by 1 min rest phases, on a Kin Trex device at 60°/s angular velocity. These exercise sessions were separated by a period of 3 weeks during which the subjects of the CG abstained from strenuous exercise while the volunteers assigned to the TG were submitted to five training sessions consisting of five stages of 10 submaximal contractions of the knee extensor and flexor muscle groups of both legs according to the above protocol, with one to two training sessions per week. The first isokinetic exercise test was followed by severe muscle pain in previously active muscles, and by significant increases in serum creatine kinase activity (SCK) and myoglobin concentration (SMb) in both groups ($p < 0.001$); those reached peak values 48 h after the exercise session. While the mean values of DOMS, SCK and SMb remained practically unchanged over time after the second isokinetic test in the CG, training was accompanied by a significant decrease of these variables ($p < 0.05$). Blood levels of interleukin-6 (IL-6) and C-reactive protein (CRP) did not change significantly over time and were not influenced by training. The hypothetical relationship between muscle damage and the production of IL-6 and CRP was not confirmed by the present results.

### IL-1β-stimulating effect on IL-6 and IL-8 productions by human chondrocytes is not mediated by nitric oxide synthase expression

Y. Henrotin,[1] S. Zheng,[1] A. Labasse,[1] P. Simonis,[1] D. Degroote[2] and J-Y. Reginster[1]
[1]Bone and Cartilage Metabolism Research Unit, University Hospital, Sart-Tilman, CHU-B23, 4000 Liège; and [2]Biosource-Medgenix S.A., B-6220 Fleurus, Belgium

Nitric oxide (NO) has been shown to be the mediator of the suppressive effect of IL-1β upon proteoglycans synthesis by chondrocytes. Moreover, NO induced chondrocytes apoptosis and inhibited cells proliferation. This study aimed to investigate the role played by NO in the IL-1β-stimulated cytokine productions by human chondrocytes. Chondrocytes were isolated from knee joint cartilage by enzymatic digestions. Chondrocytes were plated at 2 $10^5$ cells per 24-well plates and cultured for 48 h in the absence or in the presence of rhIL-1β at the concentration of 4 ng/ml and with or without 1 mM of L-$N^G$-monomethylarginine (L-NMA). IL-6 and IL-8 were assayed in the culture medium by specific EASIAs. NO formation was detected by $NO_2$ accumulation in the culture supernatants by Griess reaction with sodium nitrite as standard. Chondrocytes synthesized large amounts of nitric oxide (NO) following exposure to rhIL-1β. IL-1β also strongly stimulated IL-6 and IL-8 productions. Treatment of chondrocytes with L-NMA, a competitive inhibitor of NO synthase, inhibited both spontaneous and IL-1β-stimulated NO production but did not significantly modify cytokine productions.

| $a \neq b \neq c$, $p < 0.05$ | Controls | +L-NMA |
|---|---|---|
| DNA (μg/ml) | 1.36 ± 0.26a | 1.28 ± 0.28a |
| $NO_2$ (μg/μg DNA) | 0.36 ± 0.045a | 0.21 ± 0.045b |
| IL-6 (pg/μg DNA) | 12.5 ± 0.8a | 14.9 ± 0.5a |
| IL-8 (pg/μg DNA) | 63 ± 2a | 72 ± 12a |
| $a \neq b \neq c$, $p < 0.05$ | + IL-1β | + IL-1β + L-NMA |
| DNA (μg/ml) | 1.19 ± 0.22a | 1.19 ± 0.22a |
| $NO_2$ (μg/μg DNA) | 0.62 ± 0.094c | 0.25 ± 0.062b |
| IL-6 (pg/μg DNA) | 362 ± 12b | 305 ± 14b |
| IL-8 (pg/μg DNA) | 1190 ± 70b | 970 ± 130b |

These findings suggest that endogenously synthesized NO is not the mediator of the IL-1β-stimulating effect on IL-6 and IL-8 productions.

### Ceftazidime protects human endothelial cells from anoxia-reoxygenation damages

M. Mathy-Hartert, G. Deby-Dupont, C. Deby, M. Mouithys-Mickalad, A. Vandenberghe, L. Jadoul and M. Lamy
Centre for the Biochemistry of Oxygen, University of Liège; and Glaxo-Welcome SA, Belgium

We have shown that the cephalosporin antibiotic ceftazidime (CAZ) inhibits lipoperoxidation, desactivates singlet oxygen and protects endothelial cells (EC) against the oxidant stress induced by stimulated leukocytes.[1] By electron spin resonance (ESR) studies, we recently demonstrated the direct trapping by CAZ of free oxygen radicals (superoxide anion and hydroxyl radical) produced by phorbol myristate acetate stimulated neutrophils. We now study the effects of CAZ on ischaemia-reperfusion (IR) syndrome, a situation which is accompanied by the production of free radicalar species. Confluent EC, from human umbilical vein, were submitted to anoxia (100% $N_2$) at 37°C for 210 min and reoxygenated for 30 min (95% air–5% $CO_2$) in Hanks' balanced salt solution buffer. CAZ was added to the cells before starting anoxia. Cytotoxicity was assessed by the $^{51}Cr$ release method and by electronic microscopic observations. CAZ was protective (with variations from one cell batch to another) in a dose-dependent manner (log regression, $r^2 = 0.93$): for CAZ concentrations of $3.10^{-5}$, $5.10^{-5}$, $10^{-4}$, $3.10^{-4}$ and $10^{-3}$ M, we observed a protection of respectively $2.8 \pm 8.1$, $43.1 \pm 5.9$, $60.1 \pm 15.1$, $90.8 \pm 5.0$ and $96.1 \pm 3.3\%$, statistically significant ($p < 0.01$) from control value (without CAZ) except for the $3.10^{-5}$ M concentration. Electronic microscopic observations confirmed the protective action of CAZ: main structures of the cells were conserved, swelling of mitochondria and lysis areas were reduced compared with control cells. CAZ can thus be considered as a potential protective agent in clinical situations of IR and organ preservation before transplantation.

### Reference

1. Mathy-Hartert M, *et al. Mediators of Inflammation* 1995; **4**: 437–443.

### Inhibition of oxidative respiratory burst of human neutrophils by sphingosine derivatives: ESR and chemiluminescence study

A. Mouithys-Mickalad,[1] G. Deby-Dupont,[1,2] M. Lamy[1,2] and C. Deby[1]
[1]Centre for the Biochemistry of Oxygen, Institut de Chimie B6a; [2]Department of Anaesthesiology and Intensive Care, B35, Domaine Universitaire du Sart Tilman, 4000 Liège, Belgium

Ten years ago, Lambeth's group reported the inhibitory effect of sphinganine, a biogenic amine, on NADPH oxidase in whole cells,[1] and ascribed it to the inhibition of protein kinase C. The inhibition of NADPH oxidase by physiological amines is of interest in relation to the regulation of $O_2^-$ generating activity. Sphingosine and its N-acetyl analogue are present in mammalian cells (especially in lipid of central nervous system) and seem to be involved in NADPH oxidase activity.

In the present work, we examined the effect of sphingosine analogues (N-acetyl and N-hexanoyl-sphingosine) on active oxygen species generated by stimulated human neutrophils (PMN) using both luminol-dependent chemiluminescence (CL) and electron spin resonance (ESR) associated to spin trapping technique using 5,5-dimethyl-1-pyrroline-N-oxide (DMPO) as spin trap. PMN ($6 \times 10^6$ cells/ml for ESR assays and $1 \times 10^6$ cells/ml for CL measurements) were stimulated by phorbol myristate acetate at an initial concentration of $10^{-6}$ M in Hank's balanced salt solution pH 7.4.

CL and ESR results clearly demonstrate that sphingosine (from 2 to $8 \times 10^{-6}$ M), $C_2$-ceramide (N-acetyl-sphingosine) and $C_6$-ceramide (N-hexanoyl-sphingosine) at $2 \times 10^{-5}$ and $2 \times 10^{-4}$ M, inhibit in a dose-dependent manner the NADPH oxidase activity by interacting with phosphatidylserine (PS) component of protein kinase C, and may function physiologically as negative effectors of this enzyme. Sphingosine exhibits more pronounced effects than its analogues. According to Bazzi *et al.*[2] it could exert its function by decreasing the availability of PS or by inhibiting the substrate phospholipid interaction, resulting in the inhibition of NADPH oxidase activity.

### References

1. Wilson, *et al. J Biol Chem* 1986; **261**: 12616–12623.
2. Bazzi, *et al. Biochem Biophys Res Comm* 1987; **146**: 203–207.

### Purification of NADH-hypothiocyanite-oxidoreductase in a commensal bacterium (Streptococcus sanguis)

P. Courtois
Laboratory of Stomatology CP 622, Free University of Brussels, route de Lennik 808, B-1070 Brussels, Belgium

The resistance of *Streptococcus sanguis*—a colonizer of dental surfaces—to hypothiocyanite ($OSCN^-$)—a product of oral peroxidases—was previously attributed to a NADH-hypothiocyanite-oxidoreductase (NHOR) activity which can reduce $OSCN^-$ into thiocyanate (Oram and Reiter, 1966; Carlsson *et al.*, 1983). This activity however had not been separated so far. Fractionation of crude extracts from *Streptococcus sanguis* NCTC 7863 strain (by ultrafiltration and anion-exchange chromatography) pointed out one fraction of 125 kDa supporting NHOR activity after native-PAGE electrophoresis and gel filtration ($n = 4$). SDS-PAGE electrophoresis provided a single protein subunit of 21.1 ± 1.2 kDa (mean ± SD, $n = 9$) suggesting that NHOR protein is a hexameric complex. Purification parameters ($n = 3$) were (mean ± SD): specific activity 20.4 ± 4.0 mU/protein mg; purification factor 132 ± 65-fold; recovery 18 ± 9.6%. *Streptococcus mutans* which was inhibited by $OSCN^-$ did not present the NHOR activity; NCTC 10449 strain lysates did not reveal the NHOR protein fraction after electrophoresis. From kinetic studies an apparent $K_m$ of 20 μM was calculated for $OSCN^-$. NADH and NADPH were both cofactors for NHOR activity with the similar $K_m$ values 36 and 29 μM respectively. Further investigations should emphasize the role of NHOR as ecological selector in the oral cavity.
